# Empowering child health: Harnessing machine learning to predict acute respiratory infections in Ethiopian under-fives using demographic and health survey insights

**DOI:** 10.1186/s12879-024-09195-2

**Published:** 2024-03-21

**Authors:** Mulugeta Hayelom Kalayou, Abdul-Aziz Kebede Kassaw, Kirubel Biruk Shiferaw

**Affiliations:** 1 Department of Health Informatics, School of Public Health, College of Medicine and Health Sciences, Wollo University, Dessie, Ethiopia; 2 Department of Medical Informatics, Institute for Community Medicine, University Medicine Greifswald, Greifswald, Germany

**Keywords:** Acute respiratory infection, Artificial intelligence, Ethiopia, FAIR, Machine learning

## Abstract

**Background:**

A dearth of studies showed that infectious diseases cause the majority of deaths among under-five children. Worldwide, Acute Respiratory Infection (ARI) continues to be the second most frequent cause of illness and mortality among children under the age of five. The paramount disease burden in developing nations, including Ethiopia, is still ARI.

**Objective:**

This study aims to determine the magnitude and predictors of ARI among under-five children in Ethiopia using used state of the art machine learning algorithms.

**Methods:**

Data for this study were derived from the 2016 Ethiopian Demographic and Health Survey. To predict the determinants of acute respiratory infections, we performed several experiments on ten machine learning algorithms (random forests, decision trees, support vector machines, Naïve Bayes, and K-nearest neighbors, Lasso regression, GBoost, XGboost), including one classic logistic regression model and an ensemble of the best performing models. The prediction ability of each machine-learning model was assessed using receiver operating characteristic curves, precision-recall curves, and classification metrics.

**Results:**

The total ARI prevalence rate among 9501 under-five children in Ethiopia was 7.2%, according to the findings of the study. The overall performance of the ensemble model of SVM, GBoost, and XGBoost showed an improved performance in classifying ARI cases with an accuracy of 86%, a sensitivity of 84.6%, and an AUC-ROC of 0.87. The highest performing predictive model (the ensemble model) showed that the child’s age, history of diarrhea, wealth index, type of toilet, mother’s educational level, number of living children, mother’s occupation, and type of fuel they used were an important predicting factor for acute respiratory infection among under-five children.

**Conclusion:**

The intricate web of factors contributing to ARI among under-five children was identified using an advanced machine learning algorithm. The child’s age, history of diarrhea, wealth index, and type of toilet were among the top factors identified using the ensemble model that registered a performance of 86% accuracy. This study stands as a testament to the potential of advanced data-driven methodologies in unraveling the complexities of ARI in low-income settings.

**Supplementary Information:**

The online version contains supplementary material available at 10.1186/s12879-024-09195-2.

## Background

Despite the fact that the child mortality rate has gradually decreased over the past few decades, preventable infectious diseases continue to demand special attention. Worldwide, the majority of deaths among children under five are caused by infectious diseases, which account for 68% of all deaths [1]. The major causes of death for children under the age of five, according to the Global Health Observatory (GHO) 2016 report, were issues associated with preterm birth, acute respiratory infections (ARI), complications related to childbirth, congenital malformations, and diarrhea [2]. Acute respiratory infection includes a variety of respiratory tract infections that can cause inflammation and discomfort. The common cold, influenza, bronchitis, and pneumonia are just a few of the illnesses that fall under this category of infections. Different pathogens, such as viruses, bacteria, and occasionally fungi, can cause ARI. According to the site of infection, there are two types of acute respiratory infections: acute upper respiratory infections and acute lower respiratory infections. The severity of lower respiratory tract infections, especially pneumonia, might vary. Chest discomfort, dyspnea, and fever are more frequently brought on by lower respiratory tract infections than by upper respiratory tract infections. Both upper and lower respiratory tract infections are identified by cough [3].

Worldwide, ARI remains to be the second most frequent cause of illness and mortality among children under the age of five. The paramount disease burden in developing nations, including Ethiopia, is still ARI [4]. It caused 16% of all deaths in 2015 and killed close to one million children under the age of five, which is more than the proportion of deaths brought on by diarrhea, malaria, and measles combined [2]. Sub-Saharan Africa and Southeast Asia accounted for roughly 90% of all ARI deaths among children under the age of five. Every year, more than 12 million children with severe ARI are admitted to hospitals around the world [5]. In Ethiopia, approximately 3.4 million children are affected by ARI each year, and Ethiopia is one of the top 15 countries with the highest ARI burdens [6]. It accounts for 18% of all deaths and kills over 40,000 children under the age of five each year [7]. The prevalence of ARI in children under the age of five varies significantly between nations and regions in particular. These variations can be linked to the mother, the kid, environmental variables, and co-morbid illnesses such as malaria, measles, and diarrhea [8].

The socioeconomic and demographic characteristics that are linked to acute respiratory infection in Ethiopia have been extensively studied in the past using conventional statistical methods [9–14]. Using cross-sectional data, several studies examined the prevalence of acute respiratory infection among children under the age of five and its determining variables in various regions of Ethiopia. The most used conventional statistical methods are often limited in identifying unusual patterns and hidden relationships in Big data. The majority of previous studies used local clinical data in a single city or town, with a sample size of fewer than 500 cases or records, and were primarily concerned with a small number of predicting factors [15–18].

In this study, we applied state-of-the-art machine learning algorithms on the nationally representative dataset to predict the significant factors of acute respiratory infection in Ethiopia. Therefore, the purpose of this work was to close the gaps by developing a prediction model, detecting risk indicators, and extracting predictive factors for further development/decision-making. In doing so, we used the widely applied method of digital object standardization and sharing approach developed by the RDA (Research Data Alliance) Data Maturity model community specifically the FAIR (Findable, Accessible, Interoperable, Reusable) RDA Data maturity model to evaluate the level of source code FAIRness attained in the process.

## Methods

### Data source

The Ethiopian Demographic and Health Survey, conducted in 2016 provided the raw data for this study. As a member of the worldwide Demographic and Health Surveys program, Ethiopia participated in the EDHS 2016 for the fourth time. The Central Statistical Agency (CSA) surveyed at the Ethiopian Federal Ministry of Health’s (EFMoH) request. The data were gathered using a standardized, previously verified questionnaire. During the survey, interviewers used tablet computers. A representative sample of over 18,008 households from 624 clusters spread throughout the nine regions in Ethiopia (Amhara, Tigray, Oromia, SNNPR, Gambela, Benshangul Gumuz, Somali, Afar and Harari) and two administrative cities (Addis Ababa and Diredawa) were selected by the EDHS team.

Participation was open to all females between the ages of 15 and 49 who had at least one child in the five years before the survey. 10,006 kids under the age of five would make up the sample size for this study. A total of 9501 under-five children were included in this study after the deletion of variables with missing values greater than 50%.

### Study variables and measurements

The outcome variable was the presence or absence of ARI in a child under the age of five, and it was coded with a value of “Zero” to denote the absence of ARI and a value of “One” to denote the presence of ARI. Cough and short rapid breathing in the last two weeks for a kid were crucial to be identified as having ARI, which was measured based on mothers’ complaints regarding the symptoms of these illnesses [11].

### Data preprocessing

There are 20 features and 9501 instances in the extracted datasets. Data preprocessing techniques such as data cleaning, data transformation, handling class imbalance, and feature selection methods were used because not all of these features are pertinent for developing a predictive ARI prediction among children under the age of five in the case of Ethiopia. Mode imputation methods for categorical data were used to fill in the missing values. After doing so, we ran a Cramer’s V correlation analysis among the variables and removed variables that had very low correlation with the outcome variable and high-class imbalance. By doing this we got rid of variables with low relevance. Due to the class imbalance in the outcome variable (ARI), we tested different resampling approaches. Resampling is a widely applied method to handle class imbalance in machine learning. There are mainly two categories, the first one is to under-sample (to reduce the number of samples in the majority class to balance it with the number of samples in the minority class) and to oversample (increasing the number of samples in the minority class to balance it with the number of samples in the majority class). We used both approaches in the quest for the appropriate method for our case.

First, we trained a baseline model with a random forest algorithm with the original imbalanced data and recorded the classification report metrics mainly positive predictive value because we want the model to perform well in predicting the minority class (positive ARI cases). Then, we tested the improvement in the metrics using the different resampling techniques. The resampling techniques tested were Random over-sampler (ROS), Synthetic minority oversampling technique (SMOTE), Near miss under-sampling (NMU), and random under-sampling (RUS). Positive predictive value was considered effective in comparing oversampling techniques because the minority group of the sample had positive values (ARI positive cases).

Based on how much a feature contributes to the performance of the ensemble, its importance was measured using the SVM, GB, and XGB ensemble models. By taking into account how each feature affects the model’s prediction, these methods determine the significance scores for each feature. Besides, we used the absolute size of coefficients in relation to each other to calculate feature importance for the SVM model and permutation feature importance for GB and XGB. Higher relevance score features were thought to be more pertinent to the prediction task. To create a smaller feature set, we prioritized the highest-ranked items according to their importance scores.

### Predictive model development

Python was used as the programming language for the analysis, while GitHub was used for version control and supplementary file access. A Cori-7 processor with 16 GB of RAM and a CPU clocked at 2.8 GHz make up the hardware setup.

To develop a model that predicts ARI among under five children in Ethiopia we applied ten machine learning models (Decision Tree, Random Forest, K-nearest neighbor, Support Vector Machine (SVM), Naive Bayes, Logistic regression, Lasso Logistic regression, XGBoost, GB, Ensemble model). Grid search was used to fine-tune the hyperparameters of each algorithm since choosing the right hyperparameters has always been a critical stage in developing machine learning models and has a significant impact on the algorithm’s performance [19–21]. All the hyperparameter tuning information is available with the code. A number of metrics, including accuracy, sensitivity, specificity, weighted F1-score, AUC-ROC, and Area Under the Precision-Recall Curve (AUPRC) were used to assess each prediction model’s performance. The data processing and analysis were done using Python and all the code generated for the current study can be accessed online on GitHub https://github.com/kirubel-Biruk-Shiferaw/Empowering-Child-Health-Harnessing-Machine-Learning-to-predict-Acute-Respiratory-Infections-in-Ethi. We trained 80% of the sample at random using 5-fold cross-validation to fine-tune the model’s parameters. To determine how well the model performed, the remaining 20% of the random sample was used.

The area under the curve (AUC) and receiver operating characteristic (ROC) curve metrics were also calculated to assess how well the model performed in differentiating between ARI-positive and ARI-negative. The capacity to predict a dichotomous result is determined by comparing sensitivity vs. specificity over a range of values using ROC curves. The AUC serves as a summary of the ROC curve and measures how well a classifier can discriminate between classes [22]. Therefore, the performance of the model in separating the positive and negative classes improves with increasing AUC [22].

To determine the significance or influence of particular features on a model’s predictions, we employed the machine learning technique known as SHAP (SHapley Additive exPlanations) feature impact approach. The SHAP values help to understand the contribution of individual features to the prediction for a particular instance and can be used to analyze the overall impact of features on the model’s output.

### Model performance evaluation

The performance of the Machine Learning algorithms in this study was assessed using five performance metrics. Accuracy, Sensitivity, Specificity, Weighted F1-score and AUC-ROC. Accuracy determines how many true positives (TP), true negatives (TN), false positives (FP), and false negatives (FN) were accurately identified and it is represented by the ratio of TP and TN predictions divided by the actual positive and negative cases. Sensitivity on the other hand checks how well the model performs in terms of classifying actual true cases as TP. It is represented by the ratio of TP values and actual positive cases. Specificity is the opposite of sensitivity in the sense that it focuses on the model’s performance in classifying actual negative cases as TN values. It is represented by the ratio of TN cases and actual negative cases. F1-Score is the harmonic mean of precision and sensitivity whereas (Area under the curve of receiver operator characteristics curve) AUC-ROC measures models’ ability to classify classes [22].

### FAIRness evaluation

The concept of FAIR was introduced in 2016 by a group of scientists to set a scientific guiding principle for scientific data/digital object management and stewardship focused on facilitating the Findability, Accessibility, Interoperability, and Reusability of scientific objects including data and code/tools [23]. One of the most challenging issues in science is reproducibility and that is even worse in studies using AI approaches mainly because of the inaccessible data and code after publishing results [24, 25]. Multiple initiatives are proposing standardized data management and the FAIR guiding principle is the widely used approach for data and code sharing. Thus, we used one of the proposed FAIR assessment tools by the RDA (Research Data Alliance) to evaluate our developed model and dataset findability, accessibility, and reusability. The Research Data Alliance is a global organization with over 12,800 members from 148 countries and is built on principles that include openness, inclusivity, and transparency in scientific research [26].

## Results

### Descriptive results of the background characteristics

Out of the 9501 study subjects 83.1% of them were rural dwellers and 51.6% of them were males. When it comes to the wealth index of the respondents’ families, around 37.9% of the participants lay in the poorest category. Around 60% of the mothers had no work. The majority, 60%, of the children in the study lay between the ages of 24 months to 59 months. (Table 1).

**Table 1:** Socio-demographic characteristics of respondents in Ethiopia from January 18 to June 27, 2016 (*N* = 9501)

Variables	Frequency	Percent
**Age of child**		
< 6 months	1,088	11.45
6–11 months	1,008	10.60
12–23 months	1,820	19.15
24–35 months	1,726	18.16
36–47 months	1,890	19.89
48–59 months	1,969	20.72
**Residence**		
Urban	1,602	16.86
Rural	7,899	83.13
**Sex of child**		
Male	4,907	51.64
Female	4,594	48.35
**Wealth Index**		
Poorest	3,601	37.90
poorer	1,580	16.62
Middle	1,272	13.38
Richer	1,131	11.90
Richest	1,917	20.17
**Mothers occupation**		
Not working	5,620	59.15
working	3,881	40.84
**Number of living children**		
1–3	4,743	49.92
4–6	3,437	36.17
Above 6	1,321	13.90

### Environmental characteristics of respondents

In this survey, 5,634 families (59.3%) got their water from improved water sources. The bulk, 7,851 (82.6%), made use of unimproved restrooms. The majority of them, 8,012 (84.3%), were cooked with wood (Table 2).

**Table 2:** Environmental characteristics of the respondents in Ethiopia from January 18 to June 27, 2016 (*N* = 9501)

Variables	Frequency (N)	Percent (%)
**Source of drinking water**		
Improved	5,634	59.29
Not improved	3,739	39.35
Others*	128	1.34
**Type of cooking fuel**		
Electricity	421	4.43
Charcoal	798	8.39
Wood	8,012	84.32
Others**	270	2.84
**Type of toilet facility**		
Improved	1,486	15.64
Not improved	7,851	82.63
Others***	164	1.72

*Other water sources: not a de jure resident, lake

**Other fuel: Kerosene, straw/shrubs/grass, agricultural crop

*** other toilet facilities: not a de jure resident, no facility/bush/field

*Improved water source, such as bottled water, a piped-in residence, a piped-in yard or plot, a public tap or standpipe, a tube well or borehole; Improved toilet facilities include composting toilets, pit latrines with slabs, vented improved pit latrines, flush to piped sewer systems, and septic tanks*

### Nutritional and co-morbid characteristics among under-five children

Out of the total participants, 8,516 (89.6%) of the kids had no history of diarrhea. 9,152 (96.3%) children were breastfed. In terms of nutritional status, 2,263 (23.8%) were stunted and 1,202 (12.65%) were wasted. The majority of children, 8,430 (88.7%), did not receive any medication for intestinal parasites in the previous six months, and 5,326 (56.0%), did not receive vitamin A during that time. (Table 3)

**Table 3:** Nutritional and co-morbid characteristics of ARI among under-five children in Ethiopia from January 18 to June 27, 2016 (*N* = 9501)

Variable	Frequency (N)	Percent (%)
**Vitamin A supplement**		
Yes	4,175	43.94
No	5,326	56.05
**Stunting**		
Normal	5,709	60.08
Moderate	1,529	16.09
sever	2,263	23.81
**Wasting**		
Normal	7,565	79.62
Moderate	734	7.72
sever	1,202	12.65
**Had diarrhea**		
Yes	985	10.36
No	8,516	89.63
**Drug for intestinal parasites**		
Yes	1,071	11.27
No	8,430	88.72
**Duration of breastfeeding**		
Ever breastfed	9,152	96.32
Never breastfed	349	3.67
**Currently breastfeeding**		
Yes	6,278	66.07
No	3,223	33.92

**Fig. 1:**
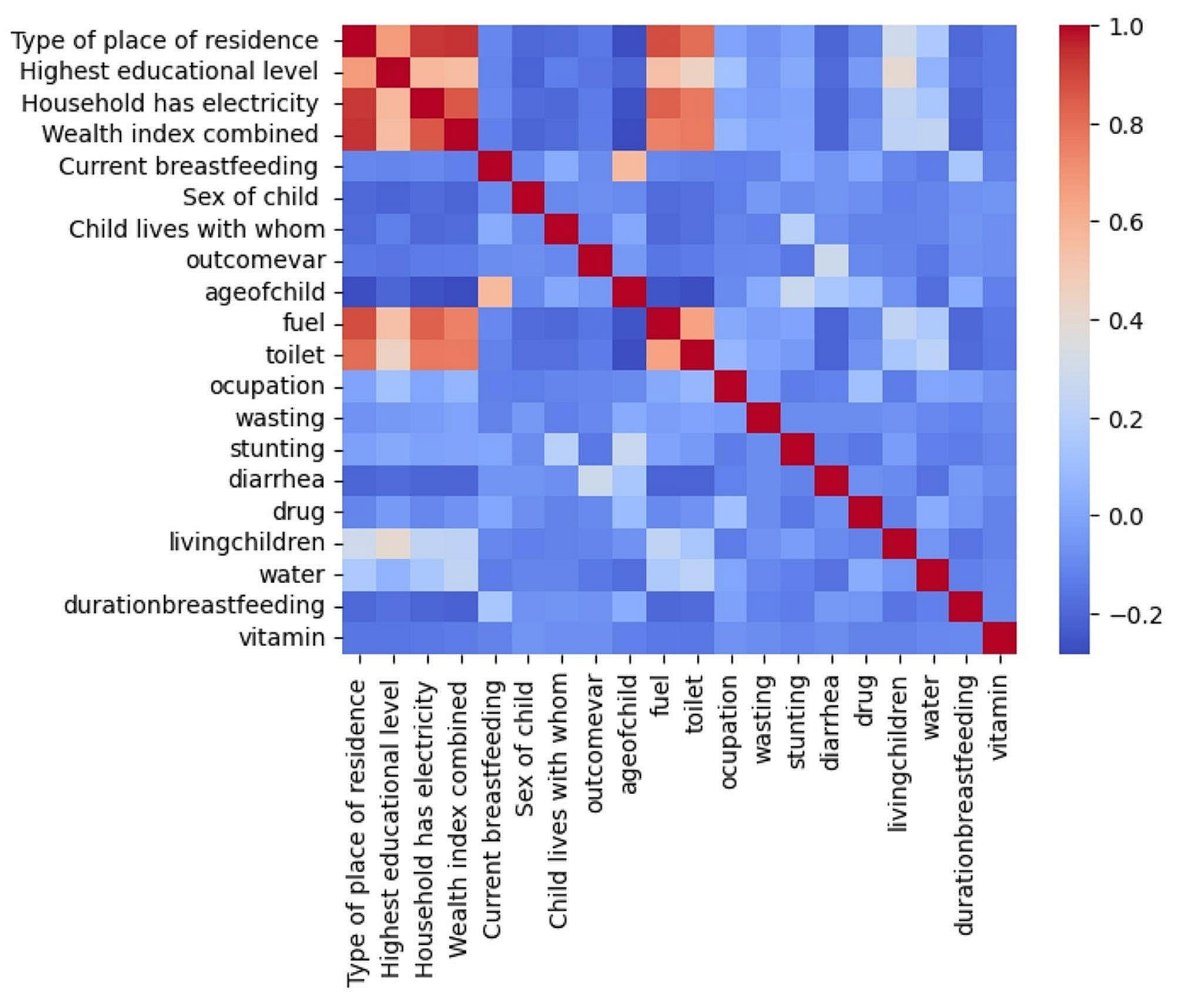
Cramer’s V Correlation heatmap

The heatmap on Fig. 1 shows that the variables have both positive and negative Cramer’s V correlations within the independent variables and also with the outcome variable. The outstanding positive correlations were observed between type of place of residence and fuel, wealth index, and education level which is not surprising and the result shows that there is no outstanding multicollinearity among the independent variables.

### Predicting under-five children’s acute respiratory infection status

Before training the models, we observed that there is a class imbalance in the outcome variable, and if not handled, will lead to biased prediction. For this reason, we first trained a baseline random forest model and computed different resampling techniques to compare improvements in model performance. Consequently, the Near miss under the sampling method resulted in better performance in classifying minority classes with improved recall value. All the results from the resampling method comparison can be found here (https://github.com/kirubel-Biruk-Shiferaw/Empowering-Child-Health-Harnessing-Machine-Learning-to-predict-Acute-Respiratory-Infections-in-Ethi). After having the resampled data, we trained the proposed ten machine learning models.

Of the ten models, SVM, GB, and XGB were the ones with the highest performance in predicting ARI cases. We further experimented with assembling best-performing models and the result from assembling SVM, GB, and XGB showed improved performance. Accuracy is not the only metric to rule out model performance; sensitivity, specificity, AUC-ROC, and AUC-PRC were also compared. The details can be found in Table 4.

**Table 4:** Performance evaluation metrics of the trained machine learning models

Machine learning algorithms										
Metrics	DT	RF	KNN	SVM	NB	LR	GB	XGB	LA	SVM + GB +XGB
**Accuracy**	0.79	0.78	0.76	0.81	0.74	0.80	0.81	0.81	0.79	0.86
**Sensitivity (%)**	74.3	79.4	62.8	78.2	52.5	74.3	76.9	85.8	73.1	84.6
**Specificity (%)**	83.3	76.9	88.4	83.3	96.1	85.8	85.8	75.6	85.8	89.7
**Weighted F1-score**	0.79	0.78	0.75	0.81	0.73	0.80	0.81	0.81	0.79	0.86
**AUC-ROC**	0.85	0.90	0.80	0.84	0.91	0.89	0.82	0.87	0.79	0.87
**AUC-PRC**	0.87	0.91	0.86	0.76	0.91	0.90	0.87	0.88	0.85	0.90

DT = Decision Tree, RF = Random Forest, KNN = K Nearest Neighbor, SVM = Support Vector Machines, NB = Naïve Bayes, LR = Logistic Regression, LA = Lasso Regression GB = Gradient Boosting, XGB = Extreme Gradient Boosting, SVM + GB + XGB = Ensemble model

**Fig. 2:**
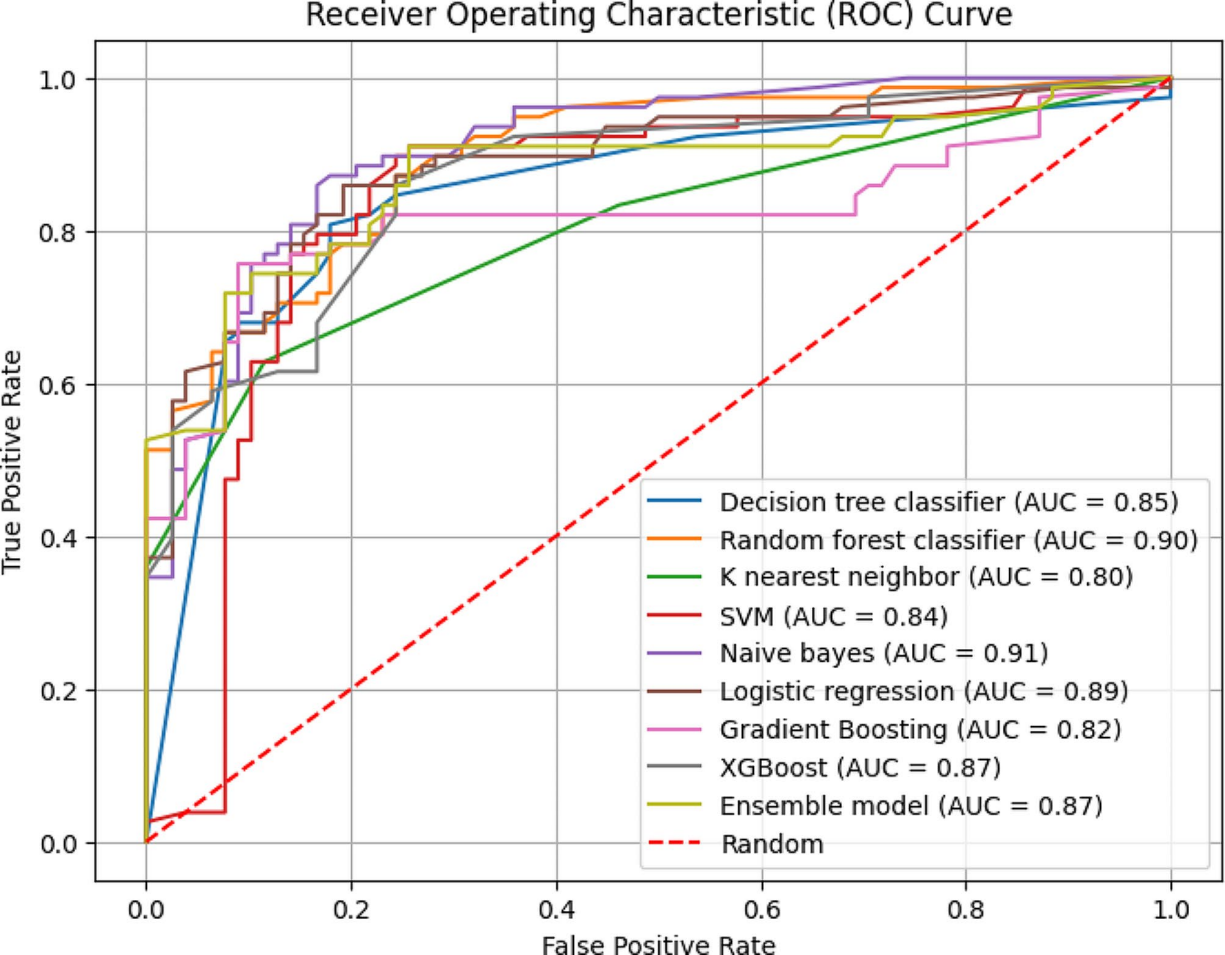
AUC-ROC values of the trained models (The comparison of training and test set AUC-ROC values are presented as a supplementary file (Appendix))

Figure 2 shows the AUC values of the trained models and to decide the best model, the tradeoff/ the threshold of accepting false positive rates was considered and the precision recall curve was further evaluated.

**Fig. 3:**
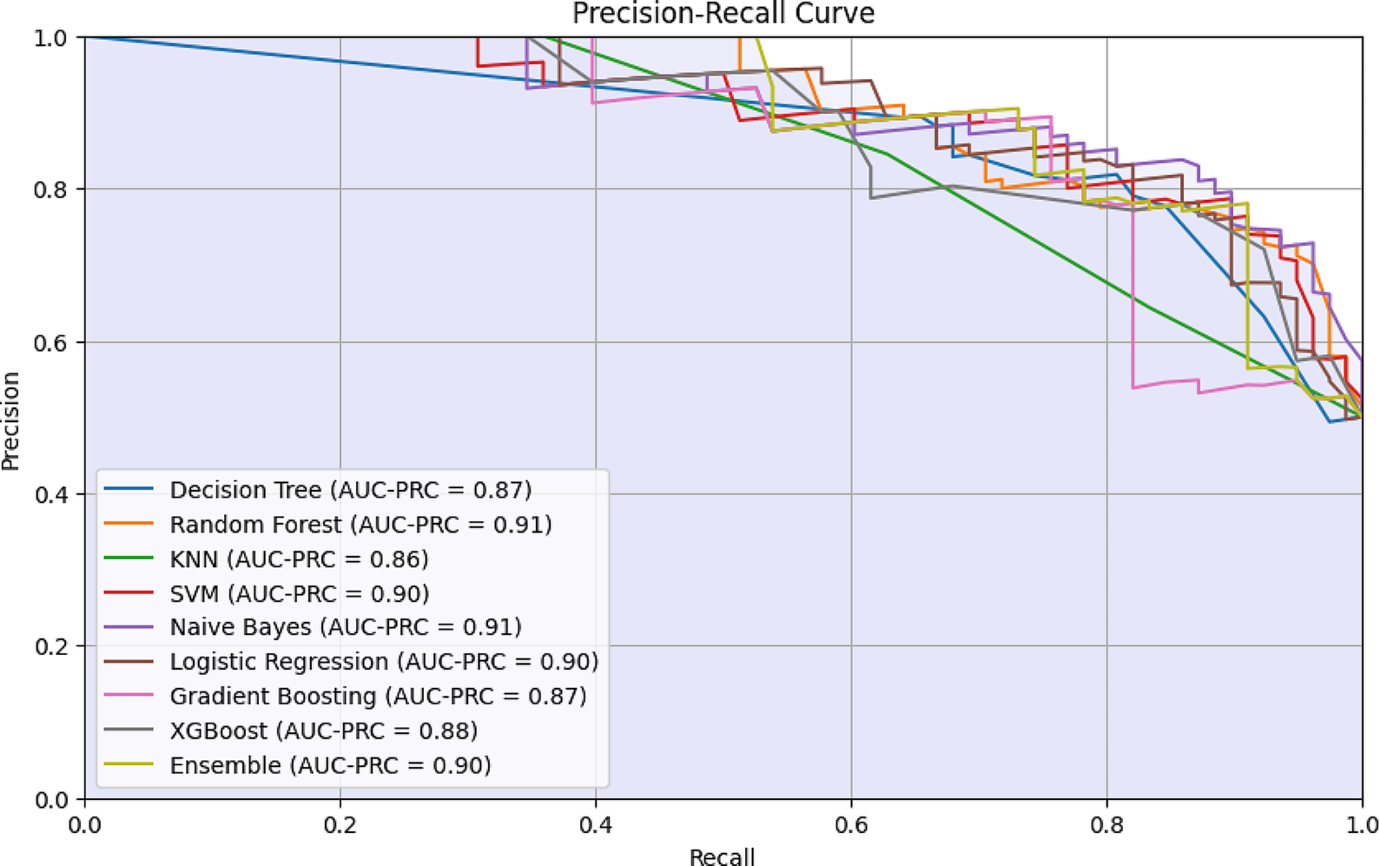
The precision-recall curve of the trained model (The comparison of AUC-PRC values for the training and test sets are presented in the supplementary file (Appendix))

The precision-recall curve is a graphical representation that shows precision-recall tradeoff for different classification thresholds. In principle, we are interested in models with higher precision and recall values but in practice, there is always a tradeoff based on the objective and priority at hand. In some cases, precision is more important than recall such as anomaly detection models and, in some cases, recall is the priority (diagnostic models). Therefore, the tradeoff between the two metrics is mainly based on the research objective and the threshold to compromise one over the other. In our case, we focus on a higher recall value for a minimum precision tradeoff which narrowed down the model selection to the ensemble model (Fig. 3).

After choosing the best performing model, which is an ensemble of SVM, GB, and XGB in our case, we assessed the learning curve to see if the model is over/underfitting, sample size/data impact and bias-variance trade-off. The result showed that the model achieved a reasonable training and validation score indicating that the model is not suffering from significant overfitting or underfitting. The result also showed that the sample size is sufficient to capture the underlying patterns which is confirmed by the cross-validation score.

### Variable importance

**Fig. 4:**
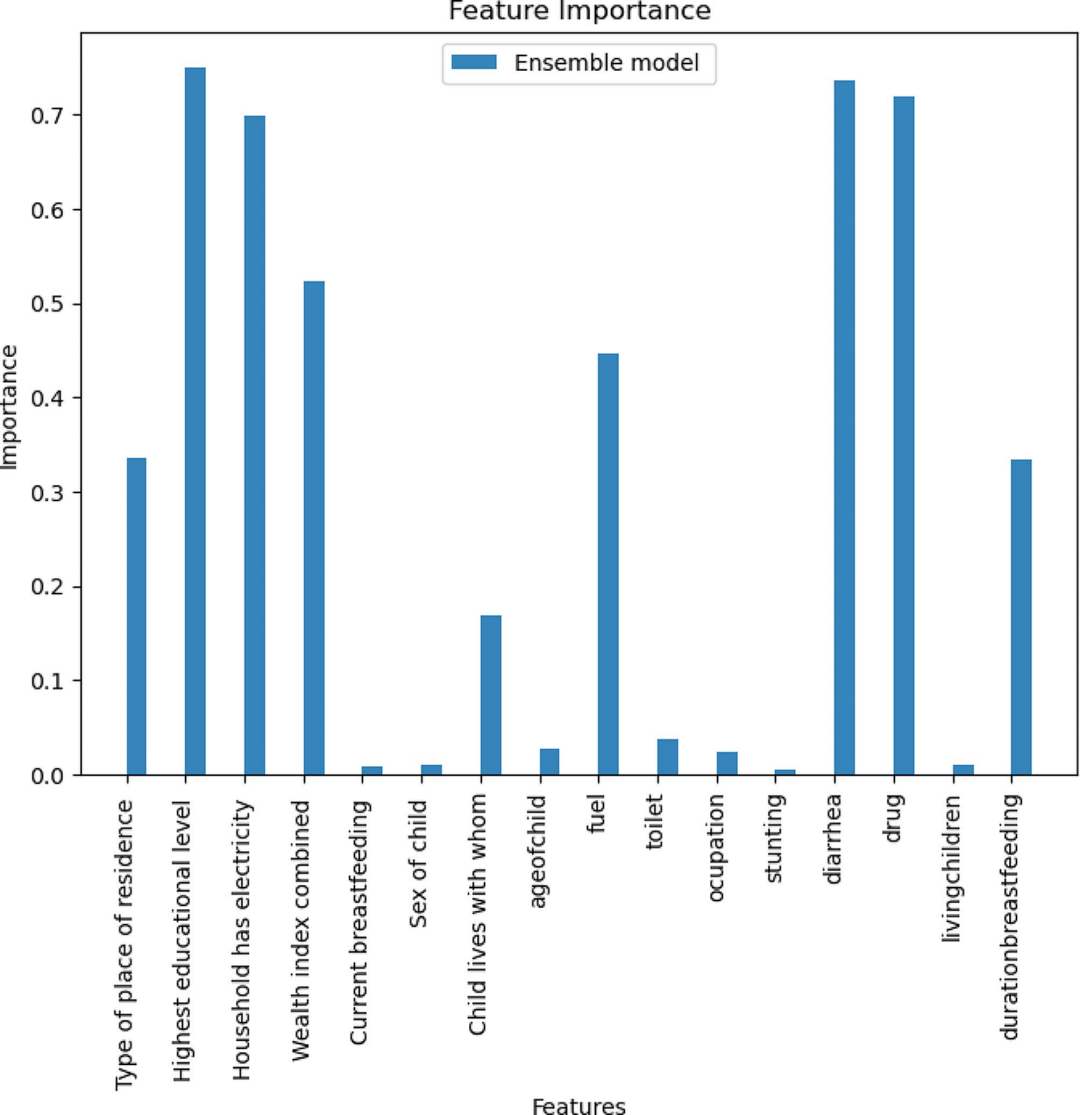
Feature importance identified by the ensemble model

Variable importance was calculated by averaging the feature importance values of the component models of the ensemble model (SVM, GB and XGB). The most important features identified by the best-performing model (ensemble model) were the highest education level, diarrhea, drug, electricity, wealth index, fuel, type of place of residence, and duration of breastfeeding. See Fig. 4 for details.

**Fig. 5:**
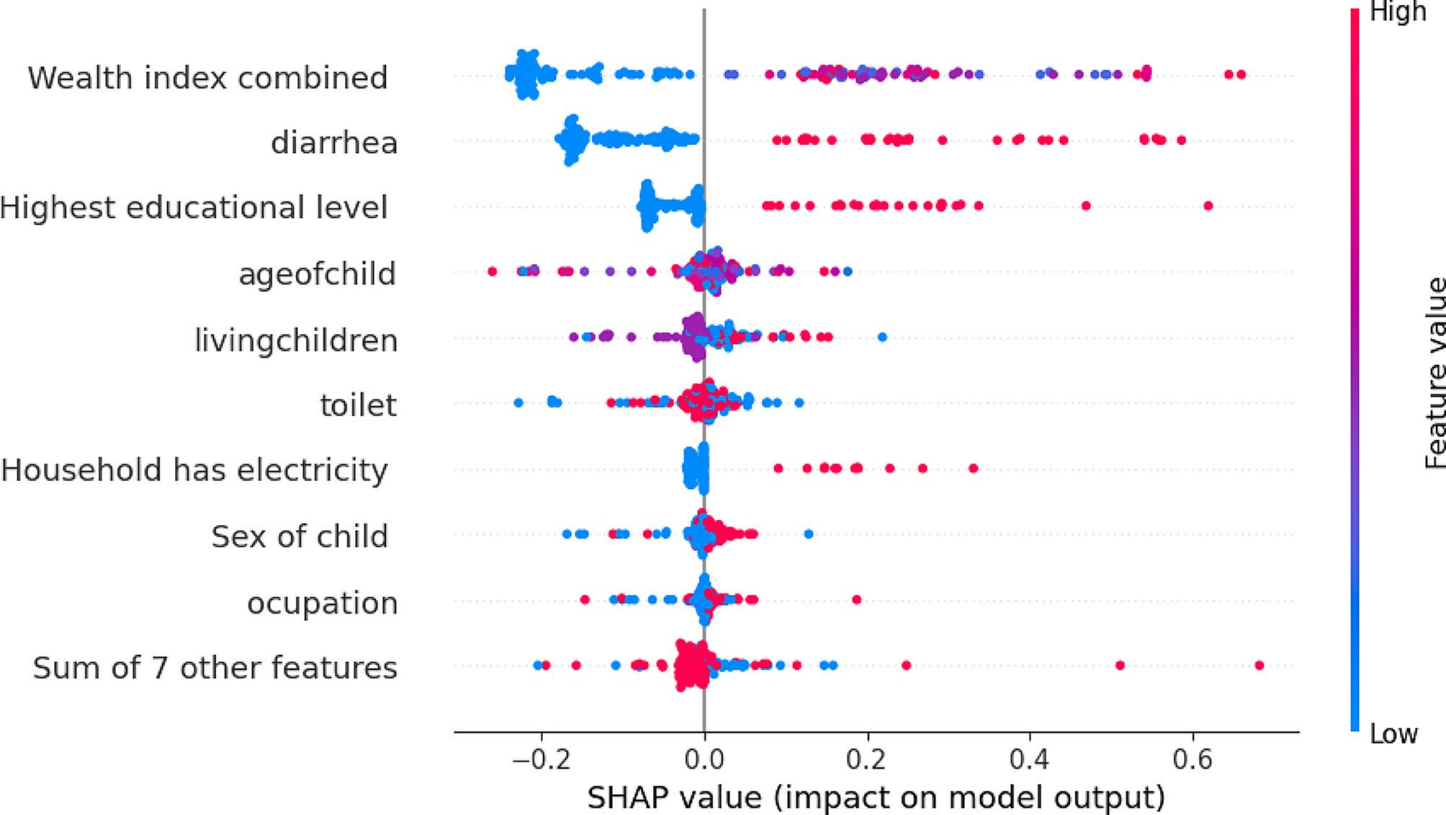
SHAP feature impact on model prediction

As shown in Fig. 5, age and toilet availability are the most important features for the negative prediction of ARI whereas wealth index, diarrhea, and education level have a low impact on the positive prediction of ARI.

### FAIRness evaluation of data and source code

**Fig. 6:**
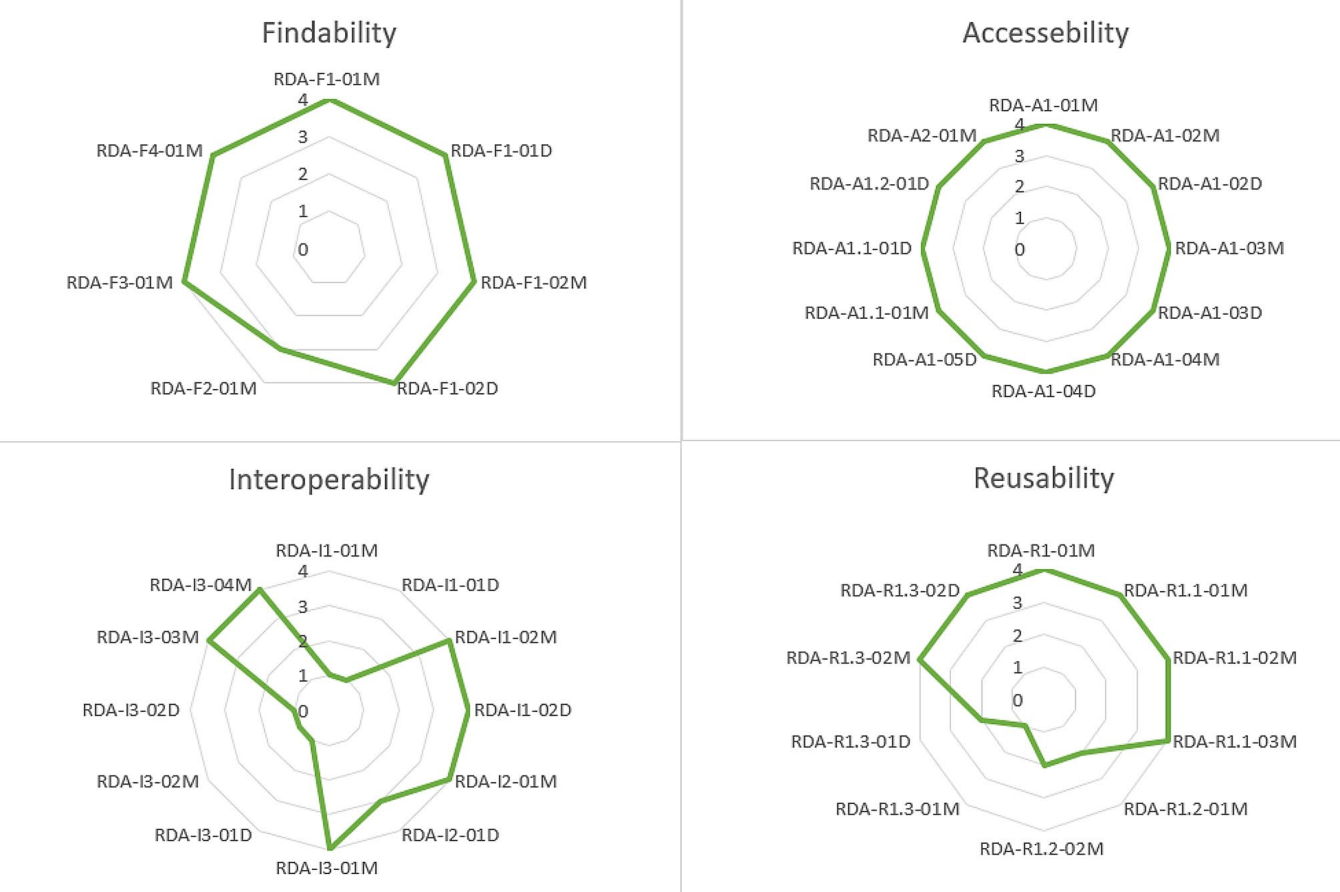
FAIR indicator assessment of data and metadata of source code. RDA = Research Data Alliance, F1-F4 = Findability indicators, A1-A2 = Accessibility indicators, D = Data, M = Metadata

The data used in this study is available on the DHS in formal request and the authors have no right to share the data publicly. However, the metadata is well documented and can be accessed (https://github.com/kirubel-Biruk-Shiferaw/Empowering-Child-Health-Harnessing-Machine-Learning-to-predict-Acute-Respiratory-Infections-in-Ethi). The RDA FAIR evaluation metrics in Fig. 6 indicated that the source code for our prediction model development satisfies all the accessibility metrics, 85.7% of findability, 50% of interoperability and 60% of reusability metrics. The calculation was done based on the requirements from the FAIR assessment tool.

## Discussion

This study presents a comprehensive analysis of factors associated with acute respiratory infection (ARI) among under-five children in Ethiopia, employing both descriptive analysis and advanced machine learning techniques. The results offer valuable insights into the socio-demographic, environmental, and nutritional dimensions of ARI risk in this vulnerable population.

### Socio-demographic insights

The socio-demographic profile of the study subjects revealed significant trends that can inform targeted interventions. The high percentage of rural dwellers (83.1%) underscores the importance of context-specific healthcare strategies for remote communities. The balanced gender distribution (51.6% males) aligns with broader gender trends in child health and points to the need for gender-sensitive healthcare policies. The considerable proportion of families categorized as poorest (37.9%) highlights the socioeconomic challenges faced by a substantial portion of the population. This finding resonates with the broader discourse on the relationship between poverty and child health outcomes.

Maternal occupation emerged as another crucial socio-demographic factor. With 60% of mothers not engaged in any work, potential implications for healthcare-seeking behavior and childcare practices warrant exploration. Additionally, the age distribution of children (60% between 24 and 59 months) corresponds to a critical developmental period. This group’s heightened susceptibility to respiratory infections necessitates targeted preventive strategies.

This study demonstrated a substantial relationship between ARI and child age. The outcome is in line with research done in the Oromia region of Ethiopia’s cities [8], where children between the ages of 2 and 11 months had a greater risk of developing ARI than those who were older. Similar to this, research from the Wondo-Genet area in southern Ethiopia [27] found that younger children (2–12 months) had a higher risk of developing ARI than older children. Children under the age of one were more likely to be hospitalized with ARI, according to other research carried out in India and the southeast of Brazil [28, 29]. It is conceivable that this is the case because young kids gradually strengthen their immune systems to fend against infectious organisms like ARI.

In this study, ARI had a greater impact on young children under the age of five who had a history of diarrhea. A case control study conducted in the Oromia zone, northeast Ethiopia, and Zimbabwe [8, 30], which found that children with a history of diarrhea were more likely to have ARI than their peers, confirmed this conclusion. Similar investigations were conducted in Ghana [31] and southwest Ethiopia [32]. According to a cross-sectional investigation conducted in Bangladesh [33], children who have a history of diarrhea are more likely to get ARI. The rationale could be that children with diarrhea who also have a concurrent sickness may have decreased immunity, rendering them more vulnerable to illnesses like ARI.

According to this study, among children under the age of five, the mother’s profession was linked to ARI. According to EDHS 2011 [34], children in Pakistan [35] who had working moms had a considerably greater chance of having ARI than children whose mothers did not work. Mothers play a significant part in childhood ARI, which might be the basis for the defense [36]. The third possibility is that working moms have been exposed to harmful chemicals, pollutants, or gases while at work, increasing the likelihood that they may infect their children. Children become more susceptible to ARI when moms are working since they do not have enough time to nurse their kids.

Additionally, this study demonstrated that the Children’s Family Wealth Index is a predictor of ARI in children under the age of five. The study conducted in Bangladesh lends weight to these conclusions [37]. Children under the age of five whose families were from lower socioeconomic classes also had a strong association with ARI [38, 39]. This is because families with more money can usually afford to provide their kids with better nutrition and healthcare. Richer households can also reduce their kids’ exposure to dangers like tainted water and unhygienic surroundings. Other studies that indicated that the risk of ARI and diarrhea is increased at increasing poverty levels provide weight to these findings.

Under-five children’s ARI was also shown to be predicted by the mother’s educational level. This assertion is in line with past discoveries [40, 41]. This could be because education has given moms the tools they need to manage their surroundings, including healthcare facilities, collaborate with medical experts more successfully, adhere to treatment recommendations, and maintain a clean environment. Furthermore, women with greater education have more influence over the health choices that their kids make.

### Environmental implications

The environmental factors investigated in this study present a complex interplay of variables that contribute to ARI risk. The majority of families relied on unimproved restrooms (82.6%) and wood for cooking (84.3%). These practices expose children to potential respiratory irritants and pollutants. These findings underscore the importance of clean cooking technologies and improved sanitation facilities to mitigate ARI risk.

### Nutritional and Health Status

Nutritional status and co-morbid factors emerged as key contributors to ARI susceptibility. Prevalence rates of stunting (23.8%) and wasting (12.65%) are alarming and signal ongoing challenges in child nutrition. Addressing these nutritional disparities is critical to reducing ARI risk and improving overall child health. The low proportion of children receiving medication for intestinal parasites (11.27%) and vitamin A supplementation (43.94%) suggests gaps in preventive healthcare measures.

### Predictive modeling for ARI

The application of machine learning models to predict ARI status yielded insightful results. Recognizing the class imbalance within the outcome variable, we adopted resampling techniques to enhance model performance. The ensemble model consisting of SVM, GB, and XGB exhibited superior predictive power, particularly in recall, which is a pivotal metric for disease detection.

The assessment of feature importance unveiled a constellation of factors influencing ARI prediction. The prominence of variables such as highest education level, diarrhea, drug usage, electricity availability, wealth index, fuel type, place of residence, and duration of breastfeeding underscores the multifaceted nature of ARI risk. The alignment of these findings with SHAP values bolsters their significance and provides a deeper understanding of the complex interplay of variables.

### Implications and future directions

The insights from this study hold implications for public health policy and interventions. Tailored strategies that address socio-demographic disparities, promote clean cooking technologies, improve sanitation, and bolster nutritional status are imperative to reducing ARI risk among under-five children. The predictive modeling framework demonstrated in this study can guide early identification and intervention efforts, thereby enhancing disease surveillance and management.

However, this study is not without limitations. The reliance on cross-sectional data restricts our ability to establish causal relationships. Additionally, the study focused on a specific geographical context and relied on a dataset from EDHS (2016), limiting generalizability. Besides, we used a single imputation method to handle missing values, that ignore uncertainty and inflated precision. Future research could explore longitudinal designs and expand the scope to broader populations with the most recent datasets.

In conclusion, this study presents a comprehensive analysis of ARI risk factors among under-five children in Ethiopia. The integration of advanced machine learning techniques enhances our understanding of these complex dynamics. The findings underscore the need for multidimensional interventions that address socio-demographic, environmental, and nutritional determinants of ARI. This study contributes to the broader discourse on child health in resource-constrained settings and offers valuable insights for evidence-based interventions.

## Conclusion

This study explored the complex landscape of acute respiratory infection (ARI) risk among children under the age of five in Ethiopia. By employing descriptive analysis and advanced machine learning techniques, this study provides a comprehensive understanding of the sociodemographic, environmental, and nutritional factors influencing ARI susceptibility. The findings highlight the need for tailored interventions that address the challenges faced by rural communities, promote gender-sensitive healthcare policies, improve environmental conditions, such as sanitation and clean cooking technologies, and tackle child malnutrition. The integration of machine learning models in ARI prediction represents a significant advancement and offers the potential for evidence-based interventions to alleviate the burden of ARI among young children in Ethiopia and similar contexts.

Overall, this study provides insights with important implications for child health policies and interventions. By considering the multifaceted determinants of ARI, including sociodemographic, environmental, and nutritional factors, along with advanced machine learning techniques, a holistic understanding of ARI risk is achieved. This research emphasizes the need for targeted interventions in various domains and lays the foundation for evidence-based approaches to mitigate ARI among children under the age of five in Ethiopia. Future studies can build on these findings to refine interventions and deepen our knowledge of child health dynamics.

### Electronic supplementary material

Below is the link to the electronic supplementary material.

Supplementary Material 1

Supplementary Material 2

## Data Availability

The data we used for this study can be accessed from the EDHS website upon request to the authority ( https://dhsprogram.com/data/dataset/Ethiopia_Standard-DHS_2016.cfm?flag=1 ), and the source code can be accessed online on GitHub https://github.com/kirubel-Biruk-Shiferaw/Empowering-Child-Health-Harnessing-Machine-Learning-to-predict-Acute-Respiratory-Infections-in-Ethi.

## Abbreviations

ARI: Acute respiratory infection
AUC: Area under the curve
ICF: International classification of functioning
KNN: K-nearest neighbor
ML: Machine learning
NP: Negative predictive
PP: Positive predictive
RF: Random forest
ROC AUC: Area Under the Curve Receiver Operator Characteristics
SVM: Support vector machine
