# Supplementary material for: Empowering child health: Harnessing machine learning to predict acute respiratory infections in Ethiopian under-fives using demographic and health survey insights
Source: BMC Infect Dis. 2024 Mar 21;24:338. doi: 10.1186/s12879-024-09195-2 (PMC10956296; doi:10.1186/s12879-024-09195-2)
Supplement: Supplementary file 1 — Supplementary Material 1 [file 12879_2024_9195_MOESM1_ESM.docx]

**Empowering Child Health: Harnessing Machine Learning to predict Acute Respiratory Infections in Ethiopian Under-Fives using Demographic and Health Survey Insights**

**A brief summary of evaluated machine learning algorithms and their basic working principle.**

**Machine learning** is a dynamic field of study in which machines are designed to mimic human intelligence by learning from the surrounding environment (Data) [1]. With this broad definition, Machine learning approaches can be classified in to three major categories based on the learning process involved.

1. Supervised ML

This approach of ML leverages the prior domain knowledge in labeling the cases and non-cases (feature annotation) [1, 2]. The “supervision” basically refers to the process where the researchers define (annotate) the outputs (classes, features) for the training. Based on the research objective, Supervised ML are grouped into regression and classification.

1. Unsupervised ML

An approach of machine learning where an algorithm attempts to sense patterns in an unlabeled dataset [3]. Given the abundance of unlabeled data, Unsupervised ML methods provide a novel insight that could sometimes be not noticed by humans and Supervised methods.

1. Reinforcement ML

In Reinforcement ML, the algorithm learns via trial and error approach by continuously interacting with a changing environment [4]. The algorithm can perceive the environment and choose an action to obtain the biggest reward value.

In this study we used a supervised ML approach to solve a classification problem. The classification problem is to identify the underlying factors between cases and non-cases of acute respiratory infection (ARI) among under five children in Ethiopia. For this, we have tested 9 algorithms and an ensemble model to figure the best model that performs well in classifying ARI cases vs non-cases and the important features responsible for the achieved level of performance.

In the section below, we will briefly explain each algorithm used in our study.

1. **Decision Tree (DT)**

DT is a supervised learning algorithm used for both classification and regression problems. The goal of this method is to create a model that predicts the value of a target variable by learning simple decision rules inferred from the data features. A tree can be seen as a piecewise constant approximation (<https://scikit-learn.org/stable/modules/tree.html>). In our case, the algorithm will try to find the best combination of rules to classify ARI cases from non-cases using the independent variables.

1. **Random Forest (RF)**

RF is basically a combination of multiple decision trees on multiple bootstrapped sub-samples of the dataset to improve the accuracy of the result.

1. **K-Nearest Neighbors (KNN)**

KNN algorithm is supervised learning classifier, which uses proximity to make classifications or predictions about the grouping of an individual data point. While it can be used for either regression or classification problems, it is typically used as a classification algorithm, working off the assumption that similar points can be found near one another. (<https://www.ibm.com/topics/knn?mhsrc=ibmsearch_a&mhq=KNN>)

1. **Support Vector Machine (SVM)**

Support Vector Machine (SVM) is another algorithm for classification and regression problems that maximizes the predictive accuracy of a model without overfitting the training data. It works under the principle to find the best line for classifying datapoints.

1. **Naive Bayes (NB)**

NB is a supervised machine learning algorithm, which is used for classification tasks, like text classification. It is also part of a family of generative learning algorithms, meaning that it seeks to model the distribution of inputs of a given class or category. Unlike discriminative classifiers, like logistic regression, it does not learn which features are most important to differentiate between classes. <https://www.ibm.com/topics/naive-bayes>

1. **Logistic Regression (LR)**

LR is a classical statistical model (also known as logit model) which is often used for classification and predictive analytics. Logistic regression estimates the probability of an event occurring based on a given dataset of independent variables.

1. **Gradient Boosting (GB)**

Gradient boosting is a machine learning technique used in regression and classification tasks, among others. It gives a prediction model in the form of an ensemble of weak prediction models, i.e., models that make very few assumptions about the data, which are typically simple decision trees. GB algorithm builds an additive model in a forward stage-wise fashion; it allows for the optimization of arbitrary differentiable loss functions. In each stage n_classes_ regression trees are fit on the negative gradient of the loss function, e.g. binary or multiclass log loss. <https://scikit-learn.org/stable/modules/generated/sklearn.ensemble.GradientBoostingClassifier.html>

1. **eXtreme Gradient Boosting (XGB)**

XGBoost, which stands for Extreme Gradient Boosting, is a scalable, distributed gradient-boosted decision tree (GBDT) machine learning library. It provides parallel tree boosting and is the leading machine learning library for regression, classification, and ranking problems.

It’s vital to an understanding of XGBoost to first grasp the machine learning concepts and algorithms that XGBoost builds upon: supervised machine learning, decision trees, ensemble learning, and gradient boosting. <https://www.nvidia.com/en-us/glossary/data-science/xgboost/>

1. **Lasso Regression (LR)**

Lasso regression is a type of linear regression that uses shrinkage. Shrinkage is where data values are shrunk towards a central point, like the mean. The lasso procedure encourages simple, sparse models (i.e. models with fewer parameters). This particular type of regression is well-suited for models showing high levels of muticollinearity or when you want to automate certain parts of model selection, like variable selection/parameter elimination. <https://scikit-learn.org/stable/modules/generated/sklearn.linear_model.Lasso.html>

The acronym “LASSO” stands for Least Absolute Shrinkage and Selection Operator.

1. **Ensemble Model (SVM, GB, XGB)**

Ensemble methods combine the predictions of several base estimators built with a given learning algorithm in order to improve generalizability / robustness over a single estimator. More generally, ensemble models can be applied to any base learner beyond trees, in averaging methods such as Bagging methods, model stacking, or Voting, or in boosting, as AdaBoost.

After evaluating the performance of the nine supervised learning algorithms, we decided to ensemble three of the best performing models to see if that makes a better classification of ARI cases and none cases in under 5 children based of the Ethiopian Demographic Health Survey (DHS) survey data. The result indicated that the ensemble of SVM, GB and XGB yield a better performance metrics than other algorithms.

**Reference**

1. Mohri, M., A. Rostamizadeh, and A. Talwalkar, *Foundations of machine learning*. 2018: MIT press.

2. Kotsiantis, S.B., I. Zaharakis, and P. Pintelas, *Supervised machine learning: A review of classification techniques.* Emerging artificial intelligence applications in computer engineering, 2007. **160**(1): p. 3-24.

3. McAlpine, E.D., P. Michelow, and T. Celik, *The Utility of Unsupervised Machine Learning in Anatomic Pathology.* American Journal of Clinical Pathology, 2021. **157**(1): p. 5-14.

4. Qiang, W. and Z. Zhongli. *Reinforcement learning model, algorithms and its application*. in *2011 International Conference on Mechatronic Science, Electric Engineering and Computer (MEC)*. 2011. IEEE.
