# Supplementary material for: Empowering child health: Harnessing machine learning to predict acute respiratory infections in Ethiopian under-fives using demographic and health survey insights
Source: BMC Infect Dis. 2024 Mar 21;24:338. doi: 10.1186/s12879-024-09195-2 (PMC10956296; doi:10.1186/s12879-024-09195-2)

**Empowering Child Health: Harnessing Machine Learning to predict Acute Respiratory Infections in Ethiopian Under-Fives using Demographic and Health Survey Insights**

Mulugeta Hayelom Kalayou^1^*, Abdul-Aziz Kebede Kassaw^1^, Kirubel Biruk Shiferaw^2^

**Comparison of AUC-ROC and AUC-PRC values between training and test datasets.**

The comparison of the AUC-ROC (Area Under the Receiver Operating Characteristic curve) is a metric that evaluates the performance of a classification model. It quantifies the ability of the model to distinguish between positive and negative classes. Accordingly, the trained models showed relatively higher AUC-ROC on the training data suggesting that the models learned the patterns and relationships effectively. Ideally, we expect the models to perform similarly as the training set and any significant gap in the metrics alarms the presence of overfitting. The result in Figure S1 from our analysis showed that there is no significant gap which shows that the models were able to distinguish classes of a new or unseen test dataset.

**Figure S1: AUC-ROC comparison on training and test datasets**


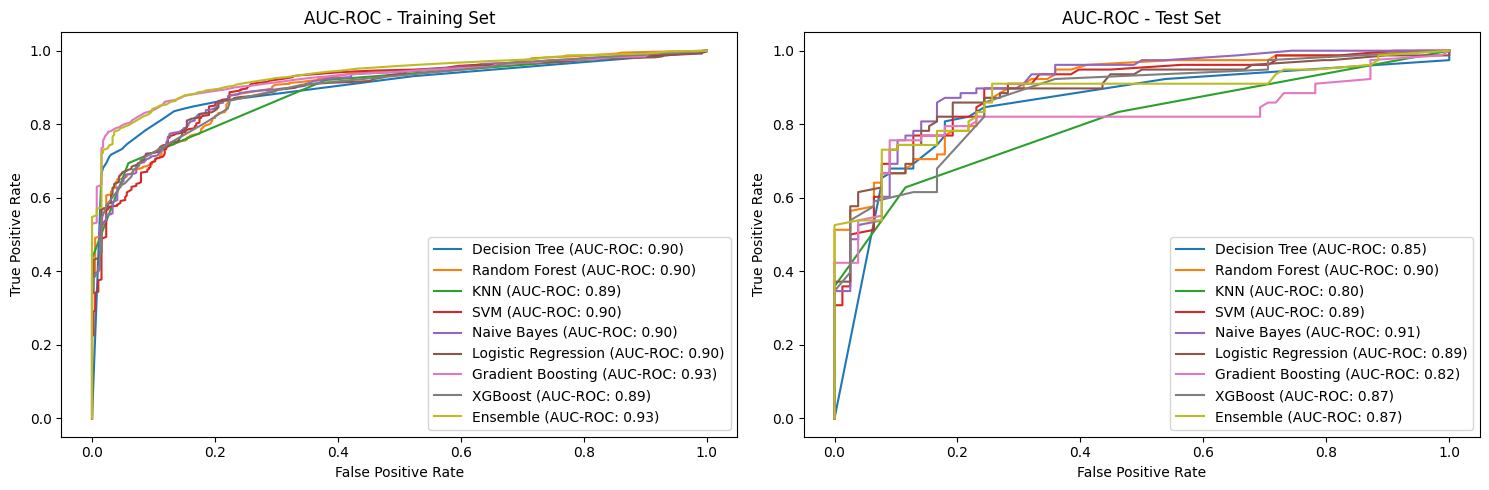


We also compared the AUC-PRC values between the training and test set. Similar to AUC-ROC, we want both the training and test values to be higher to ensure the model performance in classifying classes specially in imbalanced datasets. As the result in Figure S2 shows, we don’t have significant gap in the training and test datasets which indicates that the models are not overfitting. From this, we can deduce that the models perform well in distinguishing classes of ARI cases in imbalanced dataset.

Our best model selection is not only based on the AUC-ROC and AUC-PRC metrics but also other important metrices discussed in the manuscript.

**Figure S2: AUC-PRC comparison on training and test datasets**


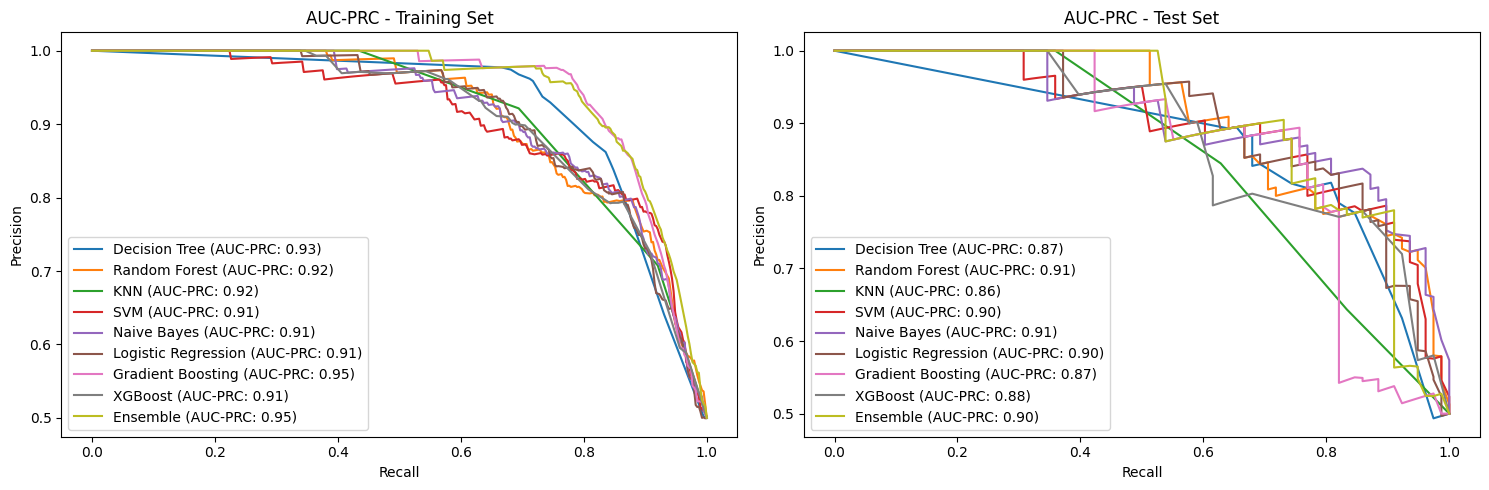

Supplement: Supplementary file 2 — Supplementary Material 2 [file 12879_2024_9195_MOESM2_ESM.docx]
